# Behavioral Skills Training for Teaching Safety Skills to Mental Health Clinicians: Protocol for a Pragmatic Randomized Control Trial

**DOI:** 10.2196/39672

**Published:** 2022-12-14

**Authors:** Elizabeth Lin, Mais Malhas, Emmanuel Bratsalis, Kendra Thomson, Rhonda Boateng, Fabienne Hargreaves, Heba Baig, Mary Benisha Benadict, Louis Busch

**Affiliations:** 1 Department of Education Centre for Addiction and Mental Health Toronto, ON Canada; 2 Department of Applied Disability Studies Brock University St. Catherines, ON Canada

**Keywords:** workplace safety, violence, mental health, medical education, protocol, occupational health, occupational safety, behavioral analysis, randomized controlled trial, RCT, pragmatic, training, safety, self protection

## Abstract

**Background:**

Workplace violence is an increasingly significant topic, particularly for staff working in mental health settings. The Centre for Addiction and Mental Health (CAMH), Canada’s largest mental health hospital, considers workplace safety a high priority and consequently has mandated staff safety training. For clinical staff, key components of this training are self-protection and team-control skills, which are a last resort when an individual is at an imminent risk of harm to self or others and other interventions are ineffective (eg, verbal de-escalation). For the past 20 years, CAMH’s training-as-usual (TAU) has been based on a 3D approach (description, demonstration, and doing), but without any competency-based assessment. Recent staff reports indicate that the acquisition and retention of these skills may be problematic and that staff are not always confident in their ability to effectively address workplace violence. The current literature lacks studies that evaluate how staff are trained to acquire these physical skills and consequently provides no recommendations or best practice guidelines. To address these gaps described by the staff and in the literature, we have used an evidence-based approach from the field of applied behavior analysis known as behavioral skills training (BST), which requires trainees to actively execute targeted skills through instruction, modeling, practice, and feedback loop. As part of this method, competency checklists of skills are used with direct observation to determine successful mastery.

**Objective:**

Our objectives are to evaluate the effectiveness of BST versus TAU in terms of staff confidence; their competence in self-protection and team-control physical skills; their level of mastery (predefined as 80% competence) in these skills; and their confidence, competency, and mastery at 1 month posttraining.

**Methods:**

We are using a pragmatic randomized controlled trial design. New staff registering for their mandatory safety training are randomly assigned to sessions which are, in turn, randomly assigned to either the BST or TAU conditions. Attendees are informed and consented into the study at the beginning of training. Differences between those consenting and those not consenting in terms of role and department are tracked to flag potential biases.

**Results:**

This study was internally funded and commenced in January 2021 after receiving ethics approval. As of May 2022, data collection is complete; half of the baseline, posttraining, and 1-month videotapes have been rated, and three-fourths of the interrater reliability checks have been completed. The analysis is expected to begin in late summer 2022 with results submitted for publication by fall 2022.

**Conclusions:**

The findings from this study are expected to contribute to both the medical education literature as well as to the field of applied behavioral analysis where randomized controlled trial designs are rare. More practically, the results are also expected to inform the continuing development of our institutional staff safety training program.

**International Registered Report Identifier (IRRID):**

DERR1-10.2196/39672

## Introduction

The promotion of health and safety in the workplace is globally recognized as a critical issue. Addressing workplace violence directly contributes to the achievement of several of the sustainable development goals set out by the International Labour Organization including health, gender equality, and decent work and economic growth [1,2]. According to the Centers for Disease Control and Prevention, workplace violence can be categorized into 4 types: criminal intent (the perpetrator has no legitimate relationship to its employees), customer or client (violence to employees from clients, family members, and visitors), worker-on-worker (horizontal violence), and personal relationship (perpetrator has a relationship to an employee outside of work setting) [3]. Globally, health care workers are at a higher risk of experiencing “customer or client” workplace violence than any other category of workers [4,5]. Two meta-analyses, representing 393,344 health care workers, demonstrated a 19.3% pooled prevalence of workplace violence in the past 12 months and 24.4% and 42.5% of respondents reported experiencing physical violence and psychological violence, respectively [6,7].

In Canada, 61% to 68% of nurses and personal support workers experienced a serious incidence of workplace violence, and 20% experienced at least 9 physical assaults in the last year [8]. In Ontario, health care workers, representing 11.7% of the province’s workforce, were also noted to be at the greatest risk of experiencing and being disproportionately affected by workplace violence compared to all other workers [9]. Within health care, psychiatric care facilities and their staff are at high risk for customer or client violence in the form of aggression from patients [10,11].

Experiencing workplace violence has been associated with negative psychological, physical, emotional, financial, and social consequences, which impact the staff’s ability to provide care and function at work [12]. More specifically, behavioral emergencies or psychiatric behaviors such as yelling, demanding, cursing, manipulating, acting out, or threatening danger are disruptive to the functioning of the unit and place the safety of everyone at risk [13].

In response, health care organizations have committed to creating a safe work environment by adopting a myriad of strategies [9]. In 2017, the Centre for Addiction and Mental Health (CAMH) adopted a major initiative to ensure the physical and psychological safety of all patients and staff. A priority component of this initiative is a mandatory training program for all new direct care staff that teaches trauma-informed crisis prevention, de-escalation skills, and, in particular, safe physical intervention skills. The physical skills curriculum focuses on self-protection and team control skills with the first set of skills targeting how staff can protect themselves when faced with physical violence and the second set related to physical restraint in the hospital. Manual restraint carries a risk of injury for all those involved and in some circumstances can provoke aggressive behavior and staff injuries [14].

The current curriculum (hereafter, “training-as-usual” [TAU]) was developed over 20 years ago. It uses the 3D approach (“describing,” “demonstrating,” and “doing” the skill through practice). However, this approach is not competency based and thus inconsistent with the contemporary directions in health care and education of using competency [15,16] and evidence-based approaches [17,18]. Furthermore, recent staff reports indicate that the skills acquired using this method are not always retained over time and that staff are not always confident about their ability to use them.

The study described in this protocol seeks to address some of these issues by evaluating 2 forms of training for all new clinical staff in a mental health care setting. We have used an evidence-based approach from the field of applied behavior analysis known as behavioral skills training (BST). BST is a competency and performance-based training model that requires trainees to accurately demonstrate targeted skills through a loop involving instruction, modeling, practice, and feedback. Checklists are used throughout the training to evaluate whether competency in a skill is achieved [19,20], and a predetermined threshold can be applied to these competency ratings to determine whether the skill has been mastered [20]. An important feature of BST is that skill rehearsal and trainer feedback are continued until the predetermined mastery criterion is achieved.

There is a large body of evidence that indicates that individuals who receive BST demonstrate significant improvement in targeted skills posttraining and maintain those skills over time and across settings [21-23]. This model has been used to train a wide range of participants including behavior analysts, parents, and educators to build safety-related skills and manage aggressive behavior [21,24].

The objective of this study is to compare the real-world effectiveness of BST and TAU in terms of physical safety skills acquisition and retention after 1 month as well as staff confidence in their use of these skills.

The following are the null hypotheses being tested: there will be no significant differences between BST and TAU in terms of (1) the competence scores or the percentage of study participants who attain mastery (predefined as a score of 80% or higher) for self-protection or team control physical skills at the end of the mandated training session; (2) the competence scores or the percentage of study participants that continue to demonstrate mastery at 1 month posttraining for either self-protection or team-control skills (retention); and (3) the percentage of study participants who express confidence in their skills at 1 month posttraining.

## Methods

### Design, Setting, and Recruitment

Because we are interested in comparing the effectiveness, rather than the efficacy, of the two training methods, we are using a pragmatic randomized controlled trial (RCT) design. The study setting is a large mental health hospital in Ontario, Canada, which delivers inpatient, outpatient, and emergency health care across a wide range of patient populations.

Newly hired staff that are classified as clinical direct service staff must undergo 2 weeks of onboarding, which includes the trauma-informed de-escalation education for safety and self-protection (TIDES) program. Within those 2 weeks, physical skills training is a full-day, in-person session scheduled on the last day. Because the onboarding is tightly scheduled, there is limited time for the typical processes of providing information about the study, gaining agreement and informed consent, and randomization. Consequently, the following procedures have been introduced.

Prior to the 2-week onboarding training, all newly hired clinical direct-service staff will be randomly assigned to their physical skills training session, and the physical skills training sessions will be randomly assigned to either BST or TAU. On the day before the physical skills session, the study will be introduced by a research team member to all attendees at the end of the last session on the previous day; during that introduction, consent will be obtained via a WebEx poll to send interested attendees a copy of the informed consent, and all attendees will be informed that a question-and-answer session along with obtaining informed consent will be carried out before the start of the physical skills session the next day, and then the informed consent form will be emailed to those who provided permission.

Just before the physical skills session, questions about the study or informed consent will be answered, and one-on-one meetings will be conducted with each attendee in a separate room to further review the informed consent and to sign indicating whether they agree or decline to participate in the study. This procedure is designed to protect against attendees identifying who is involved in the study (hereafter “study participant”) and who is not (hereafter “trainee”).

### Ethical and Safety Considerations

This study was internally funded and has been approved by the CAMH Research Ethics Board (#101-2020).

The study processes are designed to ensure that all attendees receive the same attention from the research team and facilitators and the same assessments during the training sessions. The goals are to provide equivalent training experiences, regardless of whether the person is a study participant or a trainee and regardless of whether they are in a TAU or BST session, as well as to minimize the possibility that attendees can identify who among them are or are not study participants. For example, in gaining informed consent, all attendees meet one-on-one privately with a research team member, and all sign the consent form by indicating whether they agree or decline to be study participants. In addition, skill assessments for all attendees are done individually in a separate room, but only the study participants (ie, those providing consent) are videotaped.

### Sample and Sample Size

Potential study participants include all newly hired clinical staff attending the mandatory training. There are no exclusion criteria (except the attendee’s desire not to be a study participant).

There is no published information on the expected effect size of BST versus other training methods. Indeed, most of the published BST studies have focused on efficacy and consequently have involved relatively small sample sizes. Consequently, we have opted for a sample size of 80 participants total (ie, 40 each in the BST and TAU groups) consistent with sample sizes providing 80% power for an expected medium to large effect size [25].

Recruitment began in January 2021, after REB approval was received. We reached our recruitment goal in September 2021.

### Interventions

Both the TAU and BST methods teach the same skills for protecting a person against aggression (eg, someone attempting to punch or choke the staff member) as well as to help a team of staff members physically restrain a patient who is becoming an increased risk of harm to self or others. There are 11 target skills (6 for self-protection and 5 for team-control) that are mandatory for all newly hired staff (Textbox 1). Each skill has a number of defined components, and the same sequence of steps is followed to teach each component. However, the 2 training methods differ in how these steps are administered and monitored (Table 1). The BST protocol involves attendees actively performing the targeted skills through a loop involving instruction, modeling, practice, and feedback using competency checklists [20]. This is continued until the competency of the target skill is demonstrated based on the observation of the trainer [20]. While the common practice as described in the literature is for the attendee to demonstrate successful performance of a skill 2 to 3 times before moving to the next skill on the checklist [26], we have chosen a more stringent threshold of requiring correct execution 5 times consecutively with the expectation that this will further consolidate skill acquisition and possibly retention. In contrast, TAU does not assess competency or require trainees to reach a specific level of competence for a skill before moving on to the next skill. It does, however, include modeling, practice, and feedback.

Each training session is run by 2 facilitators, each training half of the attendees to make the most efficient use of the allotted time. To be compliant with COVID-19 restrictions at the time of the study on the numbers of in-person attendees (maximum of 10 individuals including the trainer), 1 trainer is present at the session, while the other delivers the same material virtually.

**List of 11 target skills.**

**Self-protection skills**

- Same-side push/punch/grab defense
- Cross-arm grab defense
- Roundhouse or open-handed slap defense
- Same-side grab defense
- Two-handed front choke defense
- Rear choke defense

**2-5–person team control (physical restraint) skills**

- Level 1, 2-person team control
- Level 2, 2-person team control
- Level 3, 2-person team control
- Additional hand controls
- Anchor

**Table 1:** Comparison of training-as-usual (TAU) and behavioral skills training (BST) training steps.

Training strategy	TAU	BST
Target physical skills (self-protection and 2-5 person team-control)	Yes	Yes
Description of skill	Verbal with no structure	Verbal and written with systematic step-by-step instruction
Demonstration of skill by trainer	Yes	Yes
Practice of skill	Two stages (controlled practice and free practice)	Practice opportunities
Feedback	General feedback	Specific feedback using competency checklist
Repetition of description, demonstration, practice, and feedback	At the instructor’s discretion	Yes, the learner must demonstrate correct performance of 80%-100% of component steps 5 times consecutively before moving to train for the next skill
Use of competency checklist	No	Yes
Predetermined mastery criteria for each skill	No, attendees practice until the end of the allotted time	Yes, mastery for each skill defined as demonstrating correct execution of 80%-100% of component steps 5 times consecutively

### Variables Assessed

The variables to be assessed include skill competence, mastery, and retention; participant confidence in using each skill; the frequency of skill use in the previous month; and overall satisfaction with the training. Skill competence, mastery, and retention will be evaluated using checklists which were created guided by the considerations proposed by Stufflebeam [27] as well as through consultations with 2 coauthors who are board-certified behavior analysts (KB and LB). These are designed to evaluate the 11 mandatory skills being taught in the TIDES training program. The checklist creation process involved the following steps: (1) observing the training experts performing the skills and consulting with them; (2) having the organizational experts and trainers define and agree to the written descriptions in the training curriculum of the mandatory 11 target skills; (3) presenting the checklist drafts to key organizational stakeholders (professional practice office, union representatives, staff coaches, and executive leadership); (4) field-testing the resulting checklists in the mandatory staff training and making appropriate revisions; (5) and finally, making final edits based on the results of the interobserver agreement (IOA) assessment (please see further description of IOA below).

The questions assessing participant confidence, the frequency of skill use in the previous month, and overall satisfaction with the training are either adapted from existing questions used in the research team’s department or developed specifically for this study. Where applicable, 10-point Likert scales were used to improve accuracy.

The checklists (see self-protection checklist example, Multimedia Appendix 1) will be used during training for the BST sessions and for the baseline, posttraining, and 1-month study assessments for all study participants. On the training day, attendees will be asked individually to go to a separate room. They will be asked once to demonstrate each of the 11 skills prior to the start of training for baseline and just after the training for posttraining. Only those attendees who have consented to be study participants will have their baseline and posttraining assessments videotaped. For the 1-month follow-up, study participants will be asked to come in to have their skills assessed and videotaped. Videotaping will be done through WebEx (Cisco) and will be rated by a research team member using the predefined competency checklists. On these checklists, each skill component will be judged to be performed “correctly” or “incorrectly.” The final competency rating for each skill will be the percent of its component skills that were rated as correctly performed.

The reliability and validity of these ratings will be assessed using IOA calculations [28]. Prior to the study, raters had been trained on a test set of videotapes drawn from a previous study and had reached agreement levels of at least 90% for self-protection and team-control skills. For this study, all videotapes are rated by a primary rater. At the same time, a 30% random sample of videotapes from training months 1, 3, 4, 6, and 9 will be independently rated by a designated secondary (reliability) rater. IOA with the ratings from the primary rater will be calculated after every 2 videotapes rated by the secondary rater and, if the IOA is less than 90%, a recalibration discussion is held and recorded between the 2 raters with a third neutral rater available if needed to resolve any remaining differences. Recalibration decisions are then applied to all subsequent videotape ratings.

A predetermined threshold of 80%, consistent with clinical practice [29,30], is then applied to each competency rating to define skill mastery. Retention between posttraining and the 1-month follow up is measured in terms of whether the competency ratings changed as well as whether the percentage of study participants meeting the mastery threshold has changed.

Study participants complete self-reported evaluation forms covering descriptive information, confidence, training satisfaction, and frequency of skill use. The participants also provide information regarding their professional role and service department at CAMH. For confidentiality reasons, personal characteristics such as age and gender or sex were not provided to the research team, and, for the same reasons, we chose not to collect this information. All participants are asked to evaluate their confidence on a 0-10–point scale (0=not at all confident to 10=extremely confident) in using the 11 self-protection and team-control skills at baseline, immediately after the training, and at 1-month follow-up (Multimedia Appendix 2). All participants were asked to rate for satisfaction with the training on a 4-point scale immediately after the training and 1 month later. The number of times in the past month that self-protection or team-control skills were used is asked at baseline and 1-month posttraining. Finally, an open-ended item is provided in the posttraining assessment form for any comments that trainees want to communicate to the facilitators.

### Data Management and Statistical Analyses

Data from the competency checklists, completed by raters on the research team, and data from the self-reported evaluation completed by participants are collected via REDCap, which is CAMH’s designated web-based application for research data capture and then exported to Excel (Microsoft Corp) spreadsheets [31]. After data cleaning, these spreadsheets will be imported to a statistical software package (eg, SPSS, R) for analysis.

Testing of the 3 hypotheses will be done using repeated measures ANOVA. In addition, the study participants will be compared to all attendees in terms of their role and department to evaluate whether there are any striking differences despite our randomization methods. We also plan to assess the association between the frequency of physical skills used in the past month and our measures of skill competence, mastery, retention, confidence, and satisfaction.

## Results

As of May 2022, raw data collection is complete; half of the baseline, posttraining, and 1-month videotapes have been rated, three-fourths of the IOA checks have been completed, and data cleaning has begun. Analysis of the cleaned data is expected to begin in late summer 2022 with results submitted for publication by fall 2022.

## Discussion

Because there have been no previous comparisons between TAU, as used in our institution, and BST, the results we anticipate are based primarily on face validity—that is, a competency-based approach will be better for the acquisition and retention of both skills and mastery. The literature based on health care workers in general [32] suggests that both methods will improve staff confidence, although there are no published results indicating whether one will be superior to the other in either improving or maintaining confidence.

The primary strength of our study is the pragmatic RCT design, which supports a relatively rigorous comparison between the TAU and BST methods. In addition, the study is situated in a mental health setting and includes both nurses and other health care workers addressing 2 other recommendations by Geoffrion et al [32]. However, there are important limitations. First, we were not able, for confidentiality reasons, to collect personal information such as age, sex or gender, education, or ethnicity, which are likely important influences in learning. Second, for practical reasons, we were not able to assess retention beyond 1 month posttraining. These gaps are ones that we hope to address in future investigations of the impact of BST training.

We intend to share our findings with both scientific audiences, in terms of conference presentations and peer-reviewed articles, and administrative and clinical audiences, in terms of in-house and other presentations to health care and other community stakeholder organizations. Through these dissemination efforts, we hope to add to the evidence base in medical education by providing information on the utility of competency-based assessments in training staff under conditions where the desired target skills can be clearly defined and measured [20]. There is also the potential to explore in greater detail whether methods such as BST or TAU are more suitable for achieving competency, mastery, and retention of specific self-protection and team-control skills and skill components. Our findings should also add to the applied behavioral analysis field where the use of a pragmatic RCT design is relatively novel. From a practical standpoint, the study findings are expected to influence the ongoing development and delivery of physical skills training for staff within our own institution.

## Appendix

Multimedia Appendix 1 Competency checklist (self-protection skills example).

Multimedia Appendix 2 Confidence level assessment example.

## Abbreviations

BST: behavioral skills training
CAMH: Centre for Addiction and Mental Health
IOA: interobserver agreement
RCT: randomized controlled trial
TAU: training-as-usual
TIDES: trauma-informed de-escalation education for safety and self-protection

## Data Availability

Owing to privacy and ethical reasons, access to the supporting data is restricted to the members of the research team.
