# Supplementary material for: Behavioral Skills Training for Teaching Safety Skills to Mental Health Clinicians: Protocol for a Pragmatic Randomized Control Trial
Source: JMIR Res Protoc. 2022 Dec 14;11(12):e39672. doi: 10.2196/39672 (PMC9798261; doi:10.2196/39672)
Supplement: Multimedia Appendix 1 [file resprot_v11i12e39672_app1.docx]

**Multimedia Appendix 1.** Competency checklist (self-protection skills example).

| Self-Protection Skills | | 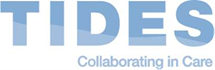 | | | |
| --- | --- | --- | --- | --- | --- |
| Training Date:_______________________________ | | ID#: | | | |
| Instructions: Please observe the learner each time they complete the skill, circle a "✔" if skills were observed and an "X" if skills were not observed. N/A if the step is not applicable | | | | |  |
|  |  |  |  |  |  |
| Attempt to Push/Grab/Choke/Punch Defense | |  | | | |
| Observed Skills | | Baseline | Post  Training | Follow-up | Comments |
| If aggressor is using right hand to attempt a push/grab/choke/punch | |  | | | |
| 1 | Step to left with left leg | ✔ X N/A | ✔ X  N/A | ✔ X  N/A |  |
| 2 | Bring both hands up to your face | ✔ X N/A | ✔ X  N/A | ✔ X  N/A |  |
| 3 | Elbows in (below your shoulders) | ✔ X N/A | ✔ X  N/A | ✔ X  N/A |  |
| 4 | Block strike with one or two forearms | ✔ X N/A | ✔ X  N/A | ✔ X  N/A |  |
| 5 | Secure aggressor's right wrist with your right hand | ✔ X N/A | ✔ X  N/A | ✔ X  N/A |  |
| 6 | Left hand on the aggressors right shoulder | ✔ X N/A | ✔ X  N/A | ✔ X  N/A |  |
| 7 | Rotate or step back with your right leg so that it is at least behind your left leg | ✔ X N/A | ✔ X  N/A | ✔ X  N/A |  |
| 8 | End up at a 45 degree angle from aggressor | ✔ X N/A | ✔ X  N/A | ✔ X  N/A |  |
| 9 | After ending up in 45 degree angle, disengage/let go within 1 second | ✔ X N/A | ✔ X  N/A | ✔ X  N/A |  |
