# Supplementary material for: Behavioral Skills Training for Teaching Safety Skills to Mental Health Clinicians: Protocol for a Pragmatic Randomized Control Trial
Source: JMIR Res Protoc. 2022 Dec 14;11(12):e39672. doi: 10.2196/39672 (PMC9798261; doi:10.2196/39672)
Supplement: Multimedia Appendix 2 [file resprot_v11i12e39672_app2.docx]

**Multimedia Appendix 2.** Confidence level assessment example.

1. If a patient started to behave aggressively towards you, how confident would you be that you could protect yourself? (please use a check mark √ to identify your response):

| Not at all confident 0 | 1 | 2 | 3 | 4 | 5 | 6 | 7 | 8 | 9 | Extremely confident  10 |
| --- | --- | --- | --- | --- | --- | --- | --- | --- | --- | --- |
|  |  |  |  |  |  |  |  |  |  |  |

1. If you needed to physically transfer or restrain an agitated patient, how confident would you be that you could do so? (please use a check mark √ to identify your response):

| Not at all confident 0 | 1 | 2 | 3 | 4 | 5 | 6 | 7 | 8 | 9 | Extremely confident  10 |
| --- | --- | --- | --- | --- | --- | --- | --- | --- | --- | --- |
|  |  |  |  |  |  |  |  |  |  |  |
